# Maßnahmen zur Bewältigung der COVID-19-Pandemie in Deutschland: nichtpharmakologische und pharmakologische Ansätze

**DOI:** 10.1007/s00103-021-03306-z

**Published:** 2021-03-31

**Authors:** Ulrike Grote, Mardjan Arvand, Simon Brinkwirth, Melanie Brunke, Udo Buchholz, Tim Eckmanns, Max von Kleist, Michaela Niebank, Bettina Ruehe, Kai Schulze, Anna Stoliaroff-Pépin, Marc Thanheiser, Lars Schaade, Dunja Said, Walter Haas

**Affiliations:** 1 Abteilung für Infektionsepidemiologie, Robert Koch-Institut, Berlin, Deutschland; 2 ÖGD Kontaktstelle: Infektionsepidemiologisches Krisenmanagement, Ausbruchsuntersuchungen und Trainingsprogramme (Fachgebiet 38), Abteilung für Infektionsepidemiologie, Robert Koch-Institut, Seestraße 10, 13353 Berlin, Deutschland; 3 Abteilung für Infektionskrankheiten, Robert Koch-Institut, Berlin, Deutschland; 4 MF Methodenentwicklung und Forschungsinfrastruktur, Robert Koch-Institut, Berlin, Deutschland; 5 Zentrum für Biologische Gefahren und Spezielle Pathogene (ZBS), Robert Koch-Institut, Berlin, Deutschland

**Keywords:** Nichtpharmakologische Maßnahmen, Kontaktbeschränkung, Alltagsmasken, Kontaktpersonennachverfolgung, Quarantäne/Isolation, Nonpharmaceutical interventions, Contact restrictions, Masks, Contact tracing, Quarantine/isolation

## Abstract

Beim ersten Auftreten des Erregers SARS-CoV‑2 im Dezember 2019 standen weder spezifische therapeutische Möglichkeiten noch ein Impfstoff zur Verfügung. Auch in Deutschland rückten deshalb nichtpharmakologische Maßnahmen zur Kontrolle der COVID-19-Pandemie in den Vordergrund. Am Robert Koch-Institut wurde eine Multikomponentenstrategie aus bevölkerungsbasierten und individuellen infektionshygienischen Maßnahmen entwickelt, die auf bestehenden Influenzapandemieplänen und generischen Planungen aufbaute. Der Beitrag erläutert die empfohlenen nichtpharmakologischen Maßnahmen und stellt die parallel entwickelten pharmakologischen Ansätze dar.

Zu den bevölkerungsbasierten Maßnahmen gehören u. a. allgemeine Kontaktbeschränkungen, die Versorgung mit Materialien für den Infektionsschutz, Veranstaltungsverbote, die Schließung von Bildungseinrichtungen und die Beschränkung des Reiseverkehrs. Zusätzlich sind individuelle infektionshygienische Maßnahmen notwendig: z. B. Einhaltung eines Mindestabstands, Reduktion von Kontakten, Tragen einer Mund-Nasen-Bedeckung sowie Einhaltung von Quarantäne und Isolierung. Die Maßnahmen im Gesundheitswesen bauen auf Empfehlungen der Kommission für Krankenhaushygiene und Infektionsprävention (KRINKO) auf und werden von den Fachgesellschaften spezifiziert und implementiert. Als pharmakologische Maßnahmen stehen mit Stand November 2020 eine antivirale Therapie mit Remdesivir und die Behandlung mit dem Glucocorticoid Dexamethason zur Verfügung. Monoklonale Antikörper sind zu diesem Zeitpunkt noch nicht zugelassen. Die therapeutische Antikoagulation wird empfohlen.

Die Empfehlungen werden kontinuierlich an die wachsende Kenntnis der Eigenschaften und Übertragungswege des Erregers angepasst. Eine große Herausforderung besteht darin, das Vertrauen der Bevölkerung in die empfohlenen Maßnahmen zu stärken. Viele Maßnahmen müssen individuell angewandt werden, um gemeinsam zu wirken.

## Abstract (en)

When the emerging novel SARS-CoV‑2 virus first appeared in December 2019, neither specific therapeutic options nor vaccinations were available. The role of nonpharmaceutical interventions (NPIs) became of central importance. At the Robert Koch Institute, a multilayer strategy consisting of population-based and individual preventive measures to control the pandemic was developed, which built upon existing influenza pandemic plans as well as generic plans. This paper explains the recommended NPIs and illustrates the pharmaceutical approaches developed in parallel.

Among others, general contact bans, providing material for infection prevention and control, ban of events, closing educational institutions, and restricting travel are counted among population-based measures. Additional individual preventive measures are necessary, e.g., keeping a minimum distance, reducing contacts, and wearing a mouth–nose covering as well as quarantine and isolation. Measures within the health system are based on recommendations of the Commission on Hospital Hygiene and Infection Protection (Kommission für Krankenhaushygiene und Infektionsprävention (KRINKO)) and specified and implemented by professional societies. Since November 2020, an antiviral therapy with remdesivir and treatment with the glucocorticoid dexamethasone have been available as pharmaceutical interventions. Monoclonal antibodies are at this time not approved. Therapeutic anticoagulation is recommended.

Recommendations are constantly adapted to the increasing knowledge on the pathogen and its means of transmission. A challenge is to strengthen the trust of the population. Many measures have to be applied on an individual basis in order to work together.

## Einleitung

Mit SARS-CoV‑2 trat zum Jahreswechsel 2019/2020 ein neuartiger Erreger auf, über den zunächst noch sehr wenige Informationen vorlagen und für den weder spezifische therapeutische Möglichkeiten noch ein Impfstoff zur Verfügung standen. Mit SARS-CoV‑1 führte zwischen den Jahren 2002 und 2003 ebenfalls ein neuartiges Coronavirus zu einem Ausbruch mit 8000 Erkrankungen in 26 Ländern und 744 Todesfällen (Fall-Verstorbenen-Anteil 9 %; [1]). Seit 2012 wurden 2500 zoonotische Erkrankungen und 866 Todesfälle (Fall-Verstorbenen-Anteil 34 %) durch das MERS-Coronavirus – insbesondere in Ländern der arabischen Halbinsel – beschrieben [2]. Trotz seiner Verwandtschaft mit diesen Erregern unterscheidet sich SARS-CoV‑2 und die durch dieses neue Virus hervorgerufene Erkrankung COVID-19 deutlich von den anderen Erregern und weiteren saisonal zirkulierenden humanen Coronaviren. Sehr rasch wurde deutlich, dass in der Bevölkerung weltweit keine Grundimmunität gegen diesen neuartigen Erreger existiert und nichtpharmakologische infektionshygienische Maßnahmen eine Schlüsselrolle in der Strategie zur Eindämmung und Verlangsamung der Ausbreitung einnehmen.

Initial konnte bei den Gegenmaßnahmen auf den Planungen im Rahmen des Nationalen Influenzapandemieplans und den Überlegungen im Rahmenkonzept für epidemisch bedeutsame Lagen aufgebaut werden, insbesondere auf den Kapiteln zu nichtpharmakologischen Maßnahmen (siehe u. a. Ergänzung zum Nationalen Pandemieplan – COVID-19 – neuartige Coronaviruserkrankung (04.03.2020); [3–5]). Mit den wachsenden Erkenntnissen zu dem neuen Erreger wurden diese Dokumente am 04.03.2020 um eine spezifische „Ergänzung zum Nationalen Pandemieplan – COVID-19“ erweitert. Es folgten noch weitere Ergänzungen der Strategie durch das Robert Koch-Institut (RKI)^1^:

- zum Multikomponentenkonzept nichtpharmakologischer Maßnahmen (19.03.2020),
- zur Mund-Nasen-Bedeckung im öffentlichen Raum als weitere Komponente zur Reduktion der Übertragungen von COVID-19 (14.04.2020),
- zu Zielen, Schwerpunktthemen und Instrumenten für den Infektionsschutz im Herbst und Winterhalbjahr (23.10.2020).

Der Beitrag mit Stand November 2020 erläutert zunächst den Hintergrund der Maßnahmen in den ersten Monaten der Pandemie aufgeteilt in 3 Teile: bevölkerungsbasierte nichtpharmakologische Maßnahmen, individuelle infektionshygienische Maßnahmen und nichtpharmakologische Maßnahmen zur Infektionsprävention im Gesundheitswesen. Anschließend gibt es einen Ausblick auf die parallel entwickelten pharmakologischen Ansätze.

## Bevölkerungsbasierte nichtpharmakologische Maßnahmen

**Figure 1:**
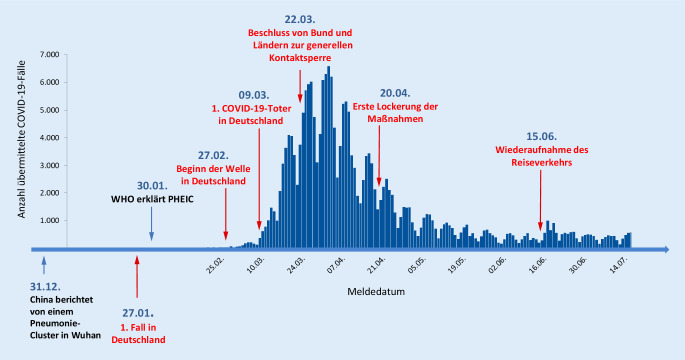

Am 27.01.2020 gab es den ersten laborbestätigten Fall von COVID-19 mit 14 Folgefällen in Deutschland (Abb. 1). Ende Februar 2020 traten weitere Cluster von COVID-19-Fällen in verschiedenen Bundesländern auf und bereits am 10.03.2020 gab es Fälle von COVID-19 in allen Bundesländern.

Mit der Zunahme von Fällen wurden verschiedene Maßnahmen ergriffen. Die Bundesländer sind selbst für die Festlegung sowie Durchführung von Maßnahmen zuständig. Dennoch waren die Maßnahmen auf Länderebene weitestgehend vergleichbar. Hierzu gehörten u. a. Maßnahmen zur Verlangsamung der Ausbreitung des Coronavirus in Deutschland (z. B. durch Kontaktbeschränkungen), die Entwicklung zusätzlicher Schutzkonzepte für besonders gefährdete Bevölkerungsgruppen und, aufgrund des beobachteten Schwereprofils, frühzeitig die Stärkung der Intensiv- und Beatmungskapazitäten in den Krankenhäusern. Gleichzeitig sollte versucht werden, den wirtschaftlichen Schaden z. B. durch Liquiditätshilfen für betroffene Unternehmen so gering wie möglich zu halten.

Im Folgenden werden die Maßnahmen, die zur Kontrolle der Fallzahlen im Frühjahr 2020 getroffen wurden, dargestellt; auf einzelne Maßnahmen in den Ländern wird nicht im Detail eingegangen.

### Allgemeine Kontaktbeschränkungen

Wegen der rasanten Ausbreitung des Virus, Ausbrüchen in Pflegeeinrichtungen und einem raschen Anstieg der Erkrankungen, die im Krankenhaus teils auf Intensivstationen behandelt werden mussten, rief die Bundesregierung die Bevölkerung bereits am 12.03.2020 dazu auf, Sozialkontakte auf das zwingend notwendige Maß zu reduzieren. Öffentliche und private Großveranstaltungen mit mehr als 1000 Teilnehmenden sollten abgesagt sowie nach Möglichkeit ganz auf Veranstaltungen verzichtet werden [6].

Um potenziell infektiöse Kontakte in Arztpraxen zu reduzieren und die Sicherheit für chronisch kranke Patientinnen und Patienten zu erhöhen, waren ab dem 11.03.2020 telefonische Krankschreibungen vorübergehend möglich.

Durch die Zunahme des Infektionsgeschehens wurden in einer weiteren Vereinbarung von Bund und Ländern am 16.03.2020 weitere Maßnahmen beschlossen: Bars, Klubs, Diskotheken, Kinos, Museen, Opern, Geschäfte etc. sollten schließen. Der Einzelhandel für Lebensmittel, Drogerien, Tankstellen, Banken und Sparkassen, Poststellen, Frisöre etc. durften unter Auflagen öffnen. Zusammenkünfte in Vereinen und sonstigen Sport- und Freizeiteinrichtungen sowie religiöse Veranstaltungen waren verboten. Restaurants sollten spätestens um 18 Uhr schließen und frühestens um 6 Uhr öffnen. Es gab Auflagen für Mensen, Gaststätten und Hotels. Übernachtungsangebote im Inland sollten nur noch zu erforderlichen und ausdrücklich nicht zu touristischen Zwecken genutzt werden [7].

Am 22.03.2020 beschlossen Bund und Länder eine Erweiterung der bisherigen Leitlinien zur Beschränkung sozialer Kontakte. Menschen mussten in der Öffentlichkeit einen Mindestabstand von 1,5 m einhalten. Es wurde ein bundesweites Versammlungsverbot eingeführt und es war nur gestattet, sich alleine, mit einer weiteren nicht im Haushalt lebenden Person oder im Kreis der Angehörigen des eigenen Hausstands in einem öffentlichen Raum aufzuhalten. Ausnahmen waren Ansammlungen von Personen, die z. B. der Versorgung mit Lebensmitteln dienten, oder die Benutzung des öffentlichen Personennahverkehrs. Zudem mussten Gastronomiebetriebe und Betriebe für die Körperpflege (Friseure, Kosmetikstudios etc.) bundesweit unverzüglich schließen [8]. Diese Maßnahmen sollten zunächst für 2 Wochen gelten; am 01.04.2020 wurde eine Verlängerung der bundesweit geltenden Kontaktbeschränkungen bis zum 19.04.2020 beschlossen [9].

### Empfehlungen für Krankenhäuser/Arztpraxen

Ein wichtiges Thema war die Sicherstellung der Versorgung mit persönlicher Schutzausrüstung und Materialien für den Infektionsschutz im medizinischen Bereich, um das Risiko nosokomialer Erkrankungen zur minimieren. So wurde aufgrund der hohen Nachfrage nach Desinfektionsmitteln von der Bundesanstalt für Arbeitsschutz und Arbeitsmedizin (BAuA) am 04.03.2020 eine Ausnahmeregelung („Allgemeinverfügung“) bekannt gegeben, die es z. B. Apotheken erlaubte, Händedesinfektionsmittel selbst herzustellen [9]. Gerade zu Beginn der Pandemie stellten medizinischer Mund-Nasen-Schutz sowie Atemschutzmasken (z. B. partikelfilternde Halbmasken wie FFP2) – neben anderen Komponenten der persönlichen Schutzausrüstung und Desinfektionsmitteln – Schlüsselressourcen für den Arbeitsschutz und die Infektionsprävention im Gesundheitswesen dar. Angesichts der begrenzten Verfügbarkeit wurden auf Anfrage des Bundesministeriums für Gesundheit (BMG) vom RKI Strategien (zeitlich begrenzt auf die voraussichtliche Dauer der Mangelsituation) für den ressourcenschonenden Einsatz in Abstimmung mit dem Bundesministerium für Arbeit und Soziales (BMAS) und dem Ad-Hoc-Arbeitskreis zu SARS-CoV‑2 des Ausschusses für Biologische Arbeitsstoffe entwickelt.

### Empfehlungen für Bildungseinrichtungen und Einrichtungenzur Kinderbetreuung

Am 12./13.03.2020 wurde von Bund und Ländern aufgrund des sich abzeichnenden dynamischen Ausbruchsgeschehens eine Verschiebung des Semesterbeginns an den Universitäten sowie eine vorübergehende Schließung von Kindergärten und Schulen in besonders betroffenen Regionen beschlossen. Die Entscheidung dazu lag hierbei jeweils in den Bundesländern. Die schrittweise Umsetzung erfolgte in der darauffolgenden Woche. Die Kindertagesbetreuungen wurden in 10 Bundesländern am 16.03.2020 geschlossen; Baden-Württemberg, Berlin und Thüringen folgten am 17.03.2020, Brandenburg, Sachsen-Anhalt und Sachsen am 18.03.2020 [10].

### Empfehlungen für den Reiseverkehr

Wegen der Ausbreitung von SARS-CoV‑2 wurde ab dem 16.03.2020 die Wiedereinführung von Grenzkontrollen zu den 5 Nachbarstaaten Österreich, Schweiz, Frankreich, Luxemburg und Dänemark eingeführt. Das Überqueren der Grenzen war nur noch Personen mit triftigem Grund, etwa Berufspendlern erlaubt. Am 17.03.2020 veröffentlichte das Auswärtige Amt eine weltweite Reisewarnung [9]. (Für weitere Informationen zu den Empfehlungen für den Reiseverkehr siehe auch den Beitrag von *Kleine-Kampmann et al.* in diesem Themenheft.)

### Lockerungen

Etwa einen Monat nach Einführung der strengen Kontaktbeschränkungen, am 20.04.2020, gab es angesichts rückläufiger Fallzahlen die ersten Lockerungen der Maßnahmen in Deutschland: In vielen Bundesländern war das Einkaufen in Geschäften bis zu einer Größe von 800 m^2^ wieder erlaubt.

In 3 Bundesländern wurde der Schulbetrieb am 20.04.2020 schrittweise wieder aufgenommen. In einer Sitzung der Jugend- und Familienministerkonferenz (JFMK) am 27.04.2020 verständigten sich die Länder auf einen gemeinsamen Rahmen für einen stufenweisen Prozess zur Öffnung der Kindertagesbetreuung, die spätestens ab dem 11.05.2020 in allen Bundesländern eingeführt werden sollte.

Am 30.04.2020 einigten sich Bund und Länder auf weitere Lockerungen der Coronaschutzmaßnahmen mit Fokus auf die Öffnung von Spielplätzen, Museen, Zoos und Gotteshäusern. Großveranstaltungen, wie z. B. Volksfeste oder größere Konzerte, blieben weiterhin untersagt. Als erstes Bundesland hob Thüringen am 13.06.2020 die Kontaktbeschränkungen auf. Es lag in der Verantwortung der Länder, über die Öffnung von Gaststätten, Beherbergungsgewerbe für touristische Zwecke, Kultureinrichtungen, Vorlesungsbetrieb an Hochschulen etc. zu entscheiden.

## Individuelle infektionshygienische Maßnahmen

Zusätzlich zu den bevölkerungsbasierten Schutzmaßnahmen wurde bereits in der ersten Strategieergänzung vom März 2020 auf die Notwendigkeit individueller infektionshygienischer Maßnahmen und die Verantwortung jedes Einzelnen in der Gesellschaft für die erfolgreiche Umsetzung der Multikomponentenstrategie hingewiesen.

Zur Prävention von Infektionen ist die Minimierung von Kontakten durch jede Einzelne und jeden Einzelnen von entscheidender Bedeutung. Frühzeitig wurden daher bundesweit Informationskampagnen zur Einhaltung eines Mindestabstands von 1,5 m gestartet und auf die Notwendigkeit der Reduktion von Kontakten auch im privaten Umfeld hingewiesen.

Am 14.04.2020 aktualisierte das RKI die Strategieergänzungen zu empfohlenen Schutzmaßnahmen und Zielen (3. Update). Hierin empfiehlt das RKI „… ein generelles Tragen einer Mund-Nasen-Bedeckung (MNB) in bestimmten Situationen im öffentlichen Raum als einen weiteren Baustein, um Risikogruppen zu schützen und den Infektionsdruck und damit die Ausbreitungsgeschwindigkeit von COVID-19 in der Bevölkerung zu reduzieren“ [11]. Sachsen führte am 20.04.2020 als Erstes im gesamten Bundesland eine Pflicht zum Bedecken von Mund und Nase im Einzelhandel und im öffentlichen Nahverkehr ein. Ab dem 29.04.2020 galt in allen Bundesländern eine MNB-Pflicht (meist für Einkäufe sowie in Bussen und Bahnen). Damit wurde nach populationsbasierten Maßnahmen wie der Schließung von Schulen und Geschäften die Bedeutung individueller, zielgerichteter nichtpharmakologischer Maßnahmen hervorgehoben.

Ab dem 15.06.2020 war zudem die Corona-Warn-App verfügbar. Diese sollte helfen, Infektionen schneller zu entdecken, und der Bevölkerung die Möglichkeit geben, vom Kontakt mit einer infizierten Person rechtzeitig zu erfahren [12]. Bei einer Stabilisierung der Fallzahlen auf niedrigem Niveau hatte die App auch das Ziel, die Kontaktpersonennachverfolgung der Gesundheitsämter zu unterstützen, um Infektionsketten zu unterbrechen und damit SARS-CoV‑2 einzudämmen.

Bereits während der ersten Welle wurde bei der Untersuchung von Ausbrüchen sowie aufgrund von experimentellen Daten die Bedeutung der Übertragung durch Aerosole auch über größere Distanzen als 1,5 m deutlich. Daher wurden für Veranstaltungen und Kontakte in Innenräumen, insbesondere auch im Hinblick auf das bevorstehende Herbst- und Winterhalbjahr, die Abstandsregeln und das Tragen von Alltagsmasken bzw. eines Mund-Nasen-Schutzes durch den Hinweis auf intensives Lüften zur Reduktion der Aerosolbelastung ergänzt: A (bstand) + H (Händehygiene) + A (Alltagsmasken) + L (üften)-Regel.

Die nachfolgenden Abschnitte widmen sich den oben genannten individuellen nichtpharmakologischen Maßnahmen und geben einen Ausblick auf die pharmakologischen Schutzmaßnahmen.

### Quarantäne und Isolierung

Quarantäne und Isolierung sind 2 Formen der Absonderung, die sich in ihrem Ansatz und ihrer Zielsetzung grundlegend unterscheiden. Empfehlungen zu ihrer Dauer erfolgen in Anpassung an den jeweils aktuellen Wissensstand.

*Der Begriff der Quarantäne bezeichnet die zeitweilige Absonderung von symptomfreien Personen, bei denen eine Ansteckung erfolgt sein kann*, da sie in Kontakt mit einer ansteckenden Person waren (Exposition). Während der Quarantäne wird die Entwicklung von Krankheitszeichen überwacht mit dem Ziel, das Risiko einer unbemerkten Infektion und damit einhergehenden Weiterverbreitung auf ein Minimum zu reduzieren. Die Dauer der Quarantäne hängt u. a. von der Inkubationszeit eines Erregers ab. Als „Inkubationszeit“ wird die Periode zwischen der Aufnahme des Infektionserregers (Ansteckung) und dem Auftreten der ersten Krankheitssymptome bezeichnet. Sie beträgt bei COVID-19 im Median 5–6 Tage (95. Perzentil bei 10–14 Tagen; [13–18]). Bei Auftreten von Symptomen oder einem positiven Erregernachweis wird die infizierte Person isoliert und somit das Risiko der Erregerverbreitung minimiert.

*Der Begriff Isolierung bezieht sich auf die Absonderung von kranken oder nachweisbar infizierten Personen.* Die Dauer der Isolierung richtet sich nach der infektiösen Periode, d. h. der Phase der Ansteckungsfähigkeit. Die Dauer der infektiösen Periode kann nur indirekt abgeleitet werden, bspw. durch die Analyse von nachweislichen Übertragungspaaren (jeweils Infektionsquelle und Folgefall mit definiertem Zeitpunkt des Kontakts) oder durch Surrogatuntersuchungen, wie bspw. der Anzüchtbarkeit des Virus im Labor. Typischerweise beginnt die Ansteckungsfähigkeit bei COVID-19 bereits 3–5 Tage nach Symptombeginn zu sinken. „Bei mild-moderater Erkrankung gilt eine Ansteckungsfähigkeit später als 10 Tage nach Symptombeginn als äußerst unwahrscheinlich und ist nur in Einzelfällen beschrieben. Bei schweren Erkrankungen oder Immunsuppression gibt es Hinweise, dass die Patienten auch noch deutlich später als 10 Tage nach Symptombeginn ansteckend sein können“ [19].

Eine Verkürzung der Quarantäne- (derzeit 14 Tage) oder Isolierungsdauer (mindestens 10 Tage, je nach Krankheitsschwere) geht grundsätzlich mit einem größeren Risiko der Ansteckung weiterer Personen einher. Andererseits können Strategien, die Quarantäne mit SARS-CoV-2-Tests kombinieren, dazu beitragen, die Nachteile einer Quarantäneverkürzung auszugleichen. Wenn sie gut konzipiert sind, können sie Vorteile für die Infektionsprävention bieten [20]. Kombinierte Quarantäne- und Teststrategien könnten daher die sozioökonomische Belastung von COVID-19 verringern.

Die Abwägung des Infektionsschutzes gegenüber anderen berechtigten sozialen, gesellschaftlichen und ökonomischen Aspekten durch eine Verkürzung von Quarantäne- und Isolierungsdauer ist auch Teil politischer Entscheidungsprozesse.

### Kontaktpersonenmanagement bei SARS-CoV-2-Infektionen

Nach einem Zusammentreffen mit einer infektiösen Person (Quellfall) kommt es – je nach Dauer und Intensität des Kontakts – im gepoolten Mittel bei ca. 4 % der engen Kontaktpersonen zu einer Infektion [21]. Bei Haushaltsmitgliedern liegt die Rate mit ca. 18 % im Mittel höher [22]. Insgesamt sind die Ergebnisse heterogen, in verschiedenen Umfeldern wurden auch deutlich höhere und niedrigere sekundäre Infektionsraten berichtet. Der Anteil der Übertragungen unterscheidet sich deutlich zwischen Haushaltskontakten, engen Kontakten außerhalb des Haushalts und sonstigen sozialen Kontakten. Bei Bekanntwerden einer Infektion des Quellfalls werden die Kontaktpersonen vom Gesundheitsamt ermittelt und über die Notwendigkeit und Dauer der Quarantäne informiert. Die Quarantäne sollte möglichst frühzeitig nach Exposition beginnen, vor Beginn der infektiösen Phase (s. unten), falls es zu einer Erkrankung oder Ausscheidung des Virus kommt. In diesem Fall würde die Infektionskette unterbrochen werden. Als „Kontaktperson“ gelten alle Personen, die 2 Tage vor bis 10 Tage nach Symptombeginn des Quellfalls Kontakt zu diesem hatten [23].

Neben dem Zeitpunkt des Kontaktes ist die Art des Kontaktes relevant: Man unterscheidet einen Kontakt mit höherem Risiko für eine Übertragung (Kontaktperson der Kategorie 1, KP1) von Kontakten mit niedrigerem Risiko (KP2). Personen der Kategorie 1 hatten entweder längeren und engeren Kontakt zu einem Quellfall (z. B. längeres Gespräch oder gemeinsames Essen) oder waren länger in einem schlecht belüfteten Raum mit einem Quellfall zusammen. Bei einem kurzen direkten engen Kontakt kann das Risiko durch das korrekte und durchgehende Tragen eines Mund-Nasen-Schutzes (MNS) oder einer Mund-Nasen-Bedeckung (MNB) reduziert werden, sodass diese Kontaktpersonen in vielen Fällen in die Kategorie 2 eingruppiert werden. Falls die Kontaktperson sich in einem Raum mit infektiösem Aerosol aufgehalten hat (z. B. Chorprobe bei schlechter Belüftung), ist ein Schutz durch MNS/MNB nicht gegeben, da diese keinen Schutz gegenüber der Aufnahme von Aerosolen gewährleisten.

Da die Inkubationszeit kurz ist, im Median 5–6 Tage, und die infektiöse Phase bereits 1–3 Tage vor Symptombeginn einsetzt, ist die Schnelligkeit der Ermittlung von Kontaktpersonen entscheidend. Der Erfolg der Kontaktpersonennachverfolgung steht und fällt somit mit der raschen Testung des Quellfalls, der zeitnahen Übermittlung des Ergebnisses und der unmittelbaren Information der Kontaktpersonen (siehe dazu auch Infobox 1). Hierdurch kann die Dauer zwischen Exposition und Quarantänebeginn so weit verkürzt werden, dass die infektiöse Periode im Falle einer Infektion der Kontaktperson noch nicht begonnen hat.

### Unterstützung der Containmentaktivitäten vor Ort mithilfe der Containment Scouts

Die zentrale Rolle zur Umsetzung des Infektionsschutzgesetzes durch das Management von Kontaktpersonen tragen die Gesundheitsämter. Um deren personelle Kapazitäten kurzfristig und möglichst effektiv zu stärken, wurde innerhalb weniger Wochen am Robert Koch-Institut (RKI) die „Containment-Scout-Initiative“ ins Leben gerufen. Ab April 2020 wurden dafür rund 500 „Containment Scouts“ (CS) bundesweit den Behörden zur Verfügung gestellt und basierend auf der Einwohnerzahl auf die verschiedenen Bundesländer aufgeteilt. Daneben sind weitere 25 „mobile“ Containment Scouts (mCS) unter Koordination des Robert Koch-Instituts bundesweit auch in anderen Gesundheitsämtern bei kurzfristigen Überlastungen unterstützend tätig.

Um die CS bestmöglich auf ihre Tätigkeit vorzubereiten, wurde ihnen zum Einstellungsbeginn Onlineschulungsmaterial zu verschiedenen Themenblöcken zur Verfügung gestellt (u. a. Kontaktpersonennachverfolgung, Meldewesen, Infektionsepidemiologie, Software zur Falldokumentation und Ausbruchsuntersuchungen). Eine unter den Gesundheitsämtern durchgeführte Umfrage deutet darauf hin, dass neben einem medizinischen Hintergrundwissen auch soziale Kompetenzen wie Motivation, Kommunikationsfähigkeiten und Stresstoleranz relevante Fähigkeiten für die Tätigkeit als CS darstellen.

Insgesamt zeigt sich, dass die CS-Iniative neben anderen Containmentaktivitäten einen wichtigen Beitrag leisten konnte. Auch ein längerfristiger Effekt der CS-Iniative zeichnet sich ab: Einige der CS mit bereits beendetem Arbeitsverhältnis wurden von den Gesundheitsämtern übernommen. Klar ist jedoch auch, dass dies nur eine vorübergehende Maßnahme ist, die eine langfristige Stärkung des Öffentlichen Gesundheitsdienstes nicht ersetzen kann.

### Mund-Nasen-Bedeckung und Mund-Nasen-Schutz in der Allgemeinbevölkerung

Als ergänzende Maßnahme zu den Hygiene- und Abstandsregelungen wurde im April 2020 das Bedecken von Mund und Nase für möglichst alle Menschen in Deutschland empfohlen. In vielen asiatischen Kulturkreisen ist es üblich, dass erkrankte Personen Masken tragen, mit dem Ziel, die Ansteckung weiterer Personen zu verhindern. Hintergrund der Empfehlung im Rahmen der COVID-19-Pandemie war die Tatsache, dass die unbemerkte Übertragung von SARS-CoV‑2 durch asymptomatisch und präsymptomatisch infizierte Personen als ein entscheidender Faktor bei der Ausbreitung des Erregers identifiziert wurde. Das Bedecken von Mund und Nase bewirkt ein Zurückhalten und ein Abbremsen der ausgestoßenen respiratorischen Tröpfchen.

Dieser abbremsende Effekt lässt sich bereits mit einfachen Materialien erreichen und benötigt nicht zwingend den Einsatz von Medizinprodukten oder einer persönlichen Schutzausrüstung. Mund-Nasen-Bedeckungen (MNB; auch „Alltagsmasken“ oder „Communitymasken“) können kommerziell und privat hergestellt und individuell gestaltet werden. Dadurch konnte die Umsetzung zeitnah und flächendeckend erfolgen, ohne die zu dem Zeitpunkt knappe Verfügbarkeit von medizinischem Mund-Nasen-Schutz (MNS) und Atemschutzmasken für das Gesundheitswesen weiter zu verschärfen.

Zu Beginn der Pandemie war die Evidenz für die Empfehlung von MNB sehr gering; die Empfehlung erfolgte daher primär aufgrund von plausiblen Ableitungen und Laboruntersuchungen. Implementiert wurde die „Maskenpflicht“ dann in Coronaschutzverordnungen auf lokaler Ebene. Die Evidenz für das Rückhaltevermögen unterschiedlicher Varianten wurde in den folgenden Monaten in zahlreichen Publikationen geschaffen, wobei sich deutlich abzeichnet, dass z. B. Visiere und andere nicht eng anliegende Bedeckungen oder der Einbau von Ausatemventilen die angestrebte Rückhaltewirkung nicht in vergleichbarer Weise erzielen [24, 25]. Die bestmögliche Rückhaltung von Tröpfchen erfolgt, wenn die MNB eng an Wangen und Kinn abschließt und durchgehend und über Mund und Nase eng anliegend getragen wird [26–28]. Auch MNS sind bei ausreichender Verfügbarkeit einsetzbar.

Voraussetzung für einen Erfolg der Gesamtmaßnahme ist, dass alle Menschen Mund und Nase situationsgerecht, konsequent und korrekt bedecken. Insbesondere gilt dies in Innenräumen bzw. wenn der Abstand nicht eingehalten werden kann. Diese Maßnahme ersetzt nicht das Einhalten des Abstandes, welcher auch für das Individuum selbst das Risiko einer Ansteckung verringert, sondern ergänzt diese im Sinne eines Gesamteffektes, der auf einem Drittschutz (Schutz der Kontaktpersonen) aller beruht aufgrund der grundsätzlichen Verringerung der Tröpfchenfreisetzung. Wichtig für die nachhaltige Akzeptanz dieser Maßnahme ist, dass die Indikationen und Ziele des Tragens von MNB von der Bevölkerung verstanden und dauerhaft umgesetzt werden.

Hinweise, dass das Bedecken von Mund und Nase mit einer MNB oder einem MNS zu gesundheitlichen Beschwerden führen könnte, liegen bisher nicht vor (Stand November 2020). Die Deutsche Gesellschaft für Pneumologie und Beatmungsmedizin e. V. (DGP) hat in einer Stellungnahme gesundheitliche Aspekte erörtert [29]. Empfehlungen für die sichere und hygienische Handhabung gibt u. a. das Bundesinstitut für Arzneimittel und Medizinprodukte (BfArM). Besonderheiten und möglichen Risiken gibt es bei der Verwendung von FFP2-Masken außerhalb des Arbeitsschutzes durch Nichtfachkundige, insbesondere durch Menschen, die einer Risikogruppe angehören (z. B. ältere Personen, Immunsupprimierte). Informationen dazu befinden sich auf der Website des RKI unter der häufig gestellten Frage (FAQ): „Ist die Verwendung von FFP2-Masken während der COVID-19-Pandemie außerhalb der Indikationen des Arbeitsschutzes sinnvoll?“ [30].

Der Erfolg dieser Maßnahme lässt sich derzeit noch nicht abschließend bewerten, jedoch liegen Hinweise und Modellierungen vor, welche einen abbremsenden Effekt auf die COVID-19-Pandemie in Deutschland nahelegen [31]. Aufgrund der Implementierung der Maskenpflicht als ein Bestandteil eines Maßnahmenbündels mit mehreren Komponenten gestaltet sich die Quantifizierung des Einzeleffektes im Bündel der Schutzmaßnahmen jedoch schwierig [32].

## Nichtpharmakologische Maßnahmen zur Infektionsprävention im Gesundheitswesen

Bereits vor dem Auftreten des ersten Falles von SARS-CoV‑2 in Deutschland hat das RKI krankenhaushygienische Maßnahmen empfohlen, welche die Prävention der Übertragung von SARS-CoV‑2 bei der Versorgung von COVID-19-Patientinnen und -Patienten im Gesundheitswesen zum Ziel hatten. Da zu Beginn der Pandemie nur wenig spezifische Kenntnisse zur Übertragung von SARS-CoV‑2 vorlagen, wurde hier zunächst in Analogie zu SARS-CoV‑1 und Influenza vorgegangen. In der nachfolgenden Zeit wurden diese Maßnahmen fortlaufend an den sich entwickelnden Kenntnisstand angepasst. Die grundlegenden Empfehlungen bauen auf den Empfehlungen der Kommission für Krankenhaushygiene und Infektionsprävention (KRINKO) auf und wurden von den jeweiligen Fachgesellschaften für bestimmte Bereiche spezifiziert und implementiert. Da es in Gesundheitseinrichtungen bestimmungsgemäß zu vielen Kontakten des Personals mit kranken Menschen bzw. Risikogruppen kommt, werden hier grundsätzlich Maßnahmen der Basishygiene umgesetzt, um generell die Übertragung von Krankheitserregern zu verhindern [33–35]. Für Maßnahmen, die über diese Basishygiene hinausgehen, müssen die grundlegenden Eigenschaften eines neuen Erregers bekannt sein, insbesondere die Übertragungswege.

Die Übertragung von SARS-CoV‑2 erfolgt auch im Gesundheitswesen primär über die respiratorische Aufnahme virushaltiger Partikel. Aerosole, also die aerogene Übertragung, spielen im klinischen Setting bei bestimmten aerosolproduzierenden Maßnahmen eine besondere Rolle. Gerade zu Beginn der Pandemie stellten daher medizinischer Mund-Nasen-Schutz sowie Atemschutzmasken – neben anderen Gegenständen der persönlichen Schutzausrüstung und Desinfektionsmitteln – Schlüsselressourcen dar.

Im Fokus stand zunächst die Abwägung von Aspekten des Infektions- und Arbeitsschutzes beim Einsatz von medizinischem MNS und Atemschutzmasken. MNS bzw. Atemschutzmasken FFP1–3 als Bestandteil der persönlichen Schutzausrüstung sind in den Empfehlungen der Bundesanstalt für Arbeitsschutz und Arbeitsmedizin (BAuA) zum Einsatz von Schutzmasken im Zusammenhang mit SARS-CoV‑2 festgehalten [36]. Entscheidend für die Infektionsprävention ist weiterhin die sachgerechte Handhabung von MNS bzw. Atemschutzmasken und die Vermeidung einer Eigenkontamination durch den Anwender [37, 38]. Mittlerweile legen Erfahrungsberichte nahe, dass bei Einhaltung der Hygienemaßnahmen eine Übertragung von SARS-CoV‑2 von Patientinnen und Patienten auf das Personal bzw. Dritte kaum stattfindet. Es kommt häufiger zu Übertragungen zwischen Beschäftigten.

Eine Übertragung von SARS-CoV‑2 über kontaminierte Oberflächen scheint in einem klinischen Setting eine untergeordnete Rolle zu spielen, wenn die routinemäßige Reinigung und Desinfektion entsprechend der KRINKO-Empfehlung „Anforderungen an die Hygiene bei der Reinigung und Desinfektion von Flächen“ stattfindet [39]. Hinsichtlich der Stabilität des Virions bzw. der Viruspartikel, deren Inaktivierung und der Bestimmung des notwendigen Wirksamkeitsbereiches wurden Analogieschlüsse aus den physikalischen Eigenschaften anderer Coronaviren, insbesondere von SARS-CoV‑1 gezogen, bevor gesicherte Erkenntnisse zur Tenazität (Haftvermögen) von SARS-CoV‑2 diese bestätigten. Die Bestimmung des erforderlichen, mindestens begrenzt viruziden Wirksamkeitsbereiches für Desinfektionsmittel erfolgte über das bestehende Schema des Arbeitskreises Viruzidie beim RKI [40].

Wie in der Allgemeinbevölkerung ist auch im Gesundheitswesen auch außerhalb der Versorgung von an COVID-19 erkrankten Personen die unbemerkte Übertragung von SARS-CoV‑2 durch infizierte, aber prä- oder asymptomatische Individuen denkbar. In Gesundheitseinrichtungen werden in der Regel vermehrt Personen mit Vorerkrankungen und Risikofaktoren behandelt, sodass hier das Risiko für eine Erkrankung sowie für einen schweren Verlauf erhöht ist. Ein weiterer Baustein, um der Übertragung von SARS-CoV‑2 entgegenzuwirken, ist das generelle Tragen von MNS durch das Personal und das gleichzeitige Tragen von MNS (oder alternativ MNB) durch die zu behandelnden Personen soweit dies toleriert wird (Double Masking), auf Basis einer einrichtungsspezifischen Risikobewertung [41, 42].

Einen Überblick über die relevantesten SARS-CoV-2-spezifischen Maßnahmen zur Patientenbetreuung im Gesundheitswesen hat das RKI bereits seit Januar 2020 zusammenfassend veröffentlicht in den „Empfehlungen des RKI zu Hygienemaßnahmen im Rahmen der Behandlung und Pflege von Patienten mit einer Infektion durch SARS-CoV-2“, welche regelmäßig aktualisiert und somit an die sich fortschreitend entwickelnde Evidenz angepasst werden.

## Pharmakologische Maßnahmen

**Figure 2:**
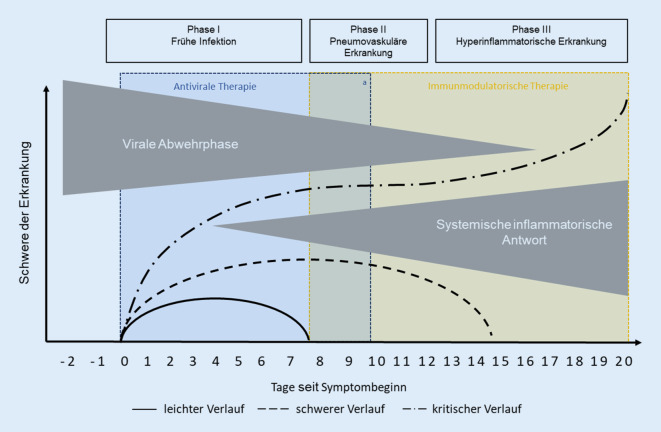

Verschiedene Arzneimittel mit direkt antiviralem oder immunmodulatorischem Wirkungsmechanismus wurden und werden im Verlauf der Pandemie in Studien untersucht. Die medikamentöse Therapie bei COVID-19 richtet sich dabei, Einzelfälle ausgenommen, orientierend nach der klinischen Phase des Krankheitsverlaufs (Abb. 2).

In der frühen Infektionsphase und teilweise noch in der frühen pulmonalen Phase der Infektion wird das klinische Bild von COVID-19 durch direkte Effekte der Virusinfektion und -replikation hervorgerufen. Bisherige Studien und Beobachtungen deuten darauf hin, dass der Zeitraum für den wirkungsvollsten Einsatz einer direkten antiviralen Therapie in der Regel höchstens die ersten 7–10 Tage nach Symptombeginn umfasst.

In der späten pulmonalen Phase sowie in der hyperinflammatorischen Phase spielen immunologische Prozesse bei der Pathogenese die wesentliche Rolle. Zu diesem Zeitpunkt stehen supportive Maßnahmen, immunmodulatorische Therapien und die Behandlung von Komplikationen im Vordergrund.

Bei Immunsupprimierten oder in Ausnahmesituationen mit anhaltendem Nachweis einer relevanten Viruslast ist eine antivirale Therapie auch später als 10 Tage nach Symptombeginn bzw. in den späteren Erkrankungsphasen im Rahmen einer Einzelfallbeurteilung zu diskutieren.

Nur wenige Arzneimittel erwiesen sich basierend auf der bisherigen Studienlage (Stand: November 2020) in speziellen Patientengruppen mit COVID-19 als wirksam.

### Antivirale Therapie mit Remdesivir

**Figure 3:**
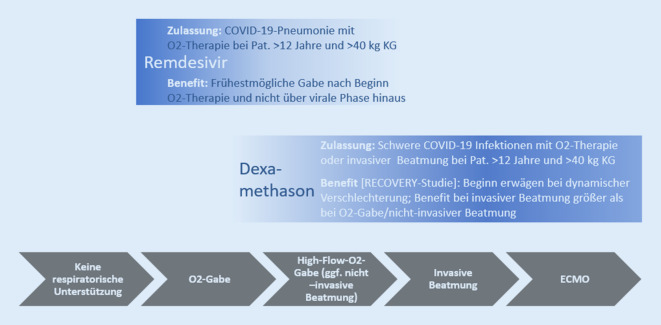

Für die Behandlung von COVID-19 bei Erwachsenen und Jugendlichen (ab einem Alter von 12 Jahren und mit einem Körpergewicht von mindestens 40 kg) mit einer Pneumonie, die eine zusätzliche Sauerstoffzufuhr erfordert, ist Remdesivir als erstes Arzneimittel zugelassen worden ([45]; Abb. 3). Bei Vorliegen einer COVID-19-Pneumonie mit erhöhtem Sauerstoffbedarf sollte die Therapie möglichst frühzeitig eingeleitet werden. Bei Patientinnen und Patienten unter nichtinvasiver oder invasiver Beatmungstherapie einschließlich extrakorporaler Membranoxygenierung (ECMO) wurde kein Nutzen gezeigt.

### Therapie mit dem Glucocorticoid Dexamethason

In einer Datenauswertung des Therapiearmes mit Dexamethason im Vergleich zur Standardbehandlung der RECOVERY-Studie konnte unter Dexamethasontherapie insgesamt eine Reduzierung der 28-Tage-Mortalität gezeigt werden ([46]; Abb. 3). Am stärksten war dieser Effekt in der Gruppe der Patientinnen und Patienten mit invasiver Beatmung und einer bei Einschluss vorliegenden Krankheitsdauer von mehr als 7 Tagen ausgeprägt.

Weniger ausgeprägt war der Effekt in der Gruppe der Patientinnen und Patienten mit Sauerstofftherapie oder nichtinvasiver Beatmung, jedoch konnte auch hier eine signifikante Reduktion der Mortalität gezeigt werden. Kein Benefit zeigte sich in der Gruppe der Patientinnen und Patienten ohne Sauerstofftherapie. [47]. Im September 2020 befürwortete die Europäische Arzneimittel-Agentur (EMA) die Erweiterung der Zulassung von Dexamethason zur Therapie bei schweren COVID-19-Verläufen [48].

### Antikörpertherapie

Die Therapie mit monoklonalen Antikörpern ist eine weitere mögliche Therapieoption (individueller Heilversuch) in der frühen (viralen) Phase von COVID-19 bzw. als Prophylaxe nach Exposition gegenüber SARS-CoV‑2. Ein Einsatz in der Frühphase der Erkrankung mit hoher Virusreplikation bei seronegativen Patientinnen und Patienten (ambulante Therapie, Postexpositionsprophylaxe) wird in aktuellen Studien untersucht. Zum jetzigen Zeitpunkt besteht noch keine Zulassung für die Antikörperbehandlung von COVID-19 in Europa. Allerdings wurde nach ersten Studienergebnissen im November 2020 von der US-amerikanischen Food and Drug Administration (FDA) eine Notfallzulassung (Emergency Use Authorization) für 2 monoklonale Antikörpertherapien ausgesprochen: für Bamlanivimab und für die Kombination aus Casirivimab und Imdevimab [49, 50].

### Antikoagulation

Als weitere spezifische pharmakologische Maßnahme wird eine frühzeitige prophylaktische Antikoagulation für den gesamten Krankheitsverlauf bei hospitalisierten Patientinnen und Patienten empfohlen und eine therapeutische Antikoagulation je nach Verlauf diskutiert [51]. Die Datenlage zum Management der Hyperkoagulabilität zur Prävention sowie zur Therapie von thromboembolischen Ereignissen ist aktuell (Stand November 2020) allerdings noch nicht einheitlich. Es konnte in einer retrospektiven Analyse sowohl für prophylaktische als auch für therapeutische Antikoagulation eine verminderte Mortalität gezeigt werden [47].

## Fazit

Die COVID-19-Pandemie belegt die Bedeutung nichtpharmakologischer Maßnahmen bei der Bewältigung einer Pandemie. Diese sind insbesondere zu Beginn einer Pandemie und während der ersten Pandemiewellen erforderlich, um die Ausbreitung des Erregers zu verlangsamen und einzudämmen. Weil bevölkerungsbasierte Maßnahmen immer auch Einschnitte in das öffentliche Leben und die Wirtschaft bedeuten, haben individuelle infektionshygienische Maßnahmen sowie die Kommunikation der Hintergründe und der richtigen Durchführung dieser Maßnahmen an die Bevölkerung einen besonderen Stellenwert. Grundlage für den Einsatz und die konkrete Umsetzung von Maßnahmen ist die rasche Verfügbarkeit von Ergebnissen aus Ausbruchsuntersuchungen und experimentellen Studien. Diese erlauben es, Schutzmaßnahmen an die Eigenschaften von SARS-CoV‑2 und seiner Übertragungswege anzupassen und weiterzuentwickeln. Eine große Herausforderung besteht darin, das Vertrauen der Bevölkerung in diese empfohlenen Maßnahmen und ihre Notwendigkeit zu stärken, da diese individuell angewandt werden, aber gemeinsam wirken. Auch pharmakologische Maßnahmen, wie Medikamente und Impfstoffe, haben einen individuellen Ansatz. Beide Ansätze sind komplementär. Nach aktuellem Stand besteht die Domäne der pharmakologischen Maßnahmen darin, im Falle einer Infektion und Erkrankung den klinischen Verlauf der Erkrankung abzumildern und Todesfälle zu verhindern. Hierbei geht der therapeutische Ansatz über die spezifische antivirale Therapie hinaus.

### Infobox 1 Die Einzelschritte der Kontaktpersonennachverfolgung können durch folgende Maßnahmen unterstützt bzw. beschleunigt werden

- Die Corona-Warn-App kann eingesetzt werden, um Kontaktpersonen zu identifizieren und die Übermittlung zu beschleunigen.
- Der Einsatz von Schnelltests ist denkbar, da hierdurch Transport- und Bearbeitungszeiten entfallen. Schnelltests haben allerdings eine niedrigere Sensitivität, sodass – im Vergleich zur PCR-Untersuchung – ein Teil der Fälle in der präsymptomatischen Phase mit noch niedriger Viruslast nicht erfasst wird.
- Wichtig ist es, die Bevölkerung in den Prozess einzubeziehen. Eine Information der Kontaktperson(en) durch den Quellfall selbst und eine freiwillige Quarantäne der Kontaktperson(en) kann Infektionsketten auch dann unterbrechen, wenn die Ermittlung durch das Gesundheitsamt nicht ausreichend zeitnah erfolgen konnte. Hierfür ist es notwendig, dass die Bevölkerung die Zusammenhänge zwischen Exposition, Inkubationszeit und Quarantäne gut verstanden hat und eine Akzeptanz auf Arbeitgeberseite für eine freiwillige Quarantäne gegeben ist.

## Footnotes

1 Siehe auch Zusammenstellung „Strategie und Krisenpläne“ auf den Coronawebseiten des RKI: https://www.rki.de/DE/Content/InfAZ/N/Neuartiges_Coronavirus/nCoV.html.
